# Nonadditivity of critical Casimir forces

**DOI:** 10.1038/ncomms11403

**Published:** 2016-04-21

**Authors:** Sathyanarayana Paladugu, Agnese Callegari, Yazgan Tuna, Lukas Barth, Siegfried Dietrich, Andrea Gambassi, Giovanni Volpe

**Affiliations:** 1 Soft Matter Lab, Department of Physics, Bilkent University, Ankara 06800, Turkey; 2 UNAM—National Nanotechnology Research Center, Bilkent University, Ankara 06800, Turkey; 3 Max-Planck-Institut für Intelligente Systeme, Heisenbergstrasse 3, D-70569 Stuttgart, Germany; 4 IV. Institut für Theoretische Physik, Universität Stuttgart, Pfaffenwaldring 57, D-70569 Stuttgart, Germany; 5 SISSA—International School for Advanced Studies and INFN, via Bonomea 265, 34136 Trieste, Italy

## Abstract

In soft condensed matter physics, effective interactions often emerge due to the spatial confinement of fluctuating fields. For instance, microscopic particles dissolved in a binary liquid mixture are subject to critical Casimir forces whenever their surfaces confine the thermal fluctuations of the order parameter of the solvent close to its critical demixing point. These forces are theoretically predicted to be nonadditive on the scale set by the bulk correlation length of the fluctuations. Here we provide direct experimental evidence of this fact by reporting the measurement of the associated many-body forces. We consider three colloidal particles in optical traps and observe that the critical Casimir force exerted on one of them by the other two differs from the sum of the forces they exert separately. This three-body effect depends sensitively on the distance from the critical point and on the chemical functionalisation of the colloid surfaces.

## Abstract (web summary)

The critical Casimir force, rising from fluctuating field confined between surfaces, is predicted to be nonadditive, but there is no experimental verification to date. Here the authors provide data support by quantifying the forces between three interacting colloidal particles using holographic traps.

## Graphical abstract

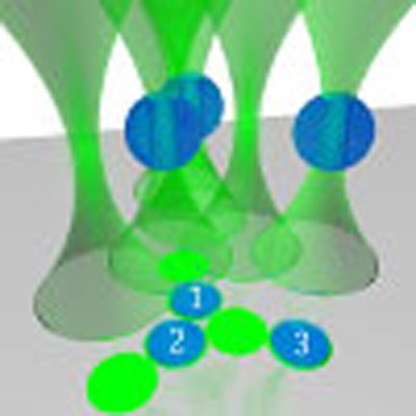

From gravitation to electromagnetism, the fundamental physical interactions are additive: for instance, the force exerted on a probe electric charge in a homogeneous medium by two other charges equals the sum of the forces exerted by each of them separately. In more complex situations, however, effective interactions take hold—and these forces are not necessarily additive^1^. Accordingly, a crucial issue is whether, and if so to what extent, genuine many-body effects are present, such as those which have been experimentally reported for the effective electrostatic interaction among charged particles immersed in a liquid medium^2^,^3^.

Effective long-ranged forces acting on mesoscopic objects emerge at the micrometre scale when these objects spatially confine a surrounding fluctuating field. Casimir forces in quantum electrodynamics (QED) are a notable example due to the confinement of vacuum electromagnetic fluctuations between two conductors^4^; these forces are typically attractive and, thus, cause undesired stiction of the metallic parts of nanodevices^5^. The thermodynamic analogues of QED Casimir forces are critical Casimir forces, which were theoretically predicted by Fisher and de Gennes in 1978 (ref. 6): the confinement of thermal fluctuations in a binary liquid mixture may result in attractive or repulsive interactions with universal features^7^. These thermal fluctuations typically occur on the molecular scale; however, on approaching a critical point of a second-order phase transition, they become relevant and correlated across a much larger (up to several microns) length scale characterized by a correlation length *ξ*. The first direct experimental evidence for critical Casimir forces was provided only in 2008 (refs 8, 9): using total internal reflection microscopy (TIRM), femtonewton effective forces were experimentally measured between a single colloid and a planar surface immersed in a critical mixture; remarkably, both attractive and repulsive forces were found, in excellent agreement with theoretical predictions. Since then, these forces have been investigated under various conditions, for example, by varying the properties of the involved surfaces^10^,^11^,^12^,^13^,^14^ or with moving boundaries^15^. In addition, a number of studies of the phase behaviour of colloidal dispersions in a critical mixture^16^,^17^,^18^,^19^,^20^,^21^ indicate critical Casimir forces as candidates for tuning the self-assembly of nanostructures^22^ and quantum dots^23^, while analogous fluctuation-induced effects have been investigated, for example, at the percolation transition of a chemical sol^24^, in the presence of temperature gradients^25^, and even in granular fluids^26^ and active matter^27^,^28^,^29^,^30^. To gain full control and possibly harness critical Casimir interactions, it is pivotal to understand to what degree the many-body effects play a role. In fact, these fluctuation-induced forces and, in particular, the critical Casimir forces are theoretically expected to be nonadditive, but experimental evidence for the corresponding many-body effects is still lacking.

Here we report a set of experiments that demonstrates and quantifies the three-body effects present in critical Casimir forces acting on colloidal microspheres immersed in a near-critical binary liquid mixture. Corresponding theoretical studies^31^,^32^,^33^ reveal that these effects can either increase or decrease the critical Casimir forces depending on the temperature *T* of the mixture, on the spatial dimensionality, on the geometrical arrangement, on the shape and on the distance between the involved surfaces, in a way that is difficult to rationalize but with an overall contribution that can be up to 20% of the pairwise additive interaction. Due to this rather complex dependence on a large number of geometrical and physical variables, it is *a priori* unclear whether this effect can be experimentally detected in colloidal suspensions. Using holographic optical tweezers (HOTs)^34^ and digital video microscopy (DVM)^35^,^36^ to probe *in situ* the forces acting on spherical colloids immersed in a critical mixture of water and 2,6-lutidine in various geometrical configurations, we observe that the critical Casimir force exerted on a probe colloid by two other colloids differs from the sum of the forces exerted by them separately. These many-body effects are controlled by adjusting the criticality of the mixture, for example, by tuning its temperature or by changing the surface properties of the particles. Since interactions among micro- and nanoscopic particles in fluids are central to a wide spectrum of physical, chemical and biological phenomena, the insight provided here might prove useful for a diverse range of applications, including control of microscopic self-assembly of colloids, formation and stabilization of nanoparticle suspensions, aggregates, colloidal molecules and photonic crystals, as well as phase and biomimetic behaviours of micro- and nanoparticles—considering in particular that the many-body effects are expected to become even more important on the nanoscale^37^,^38^.

## Results

### Outline of the experimental approach

Before getting into the experimental details, we briefly outline the strategy of the experiment. We employ a set-up in which three colloidal microspheres are held by HOTs at the corners of an almost equilateral triangle. First, we measure the two-body critical Casimir forces arising, on approaching criticality, on each of the three pairs of particles in the absence of the remaining colloid, which is temporarily moved into an auxiliary trap. Then, assuming additivity of critical Casimir forces, these measurements are used to predict the forces acting on a pair of particles in the presence of the third one, which is eventually brought closer. Finally, we compare this additive prediction with the actually measured three-body potential: the significant discrepancies that appear clearly demonstrate the nonadditivity of critical Casimir forces.

### Critical mixture and theoretical potentials

**Figure 1:**
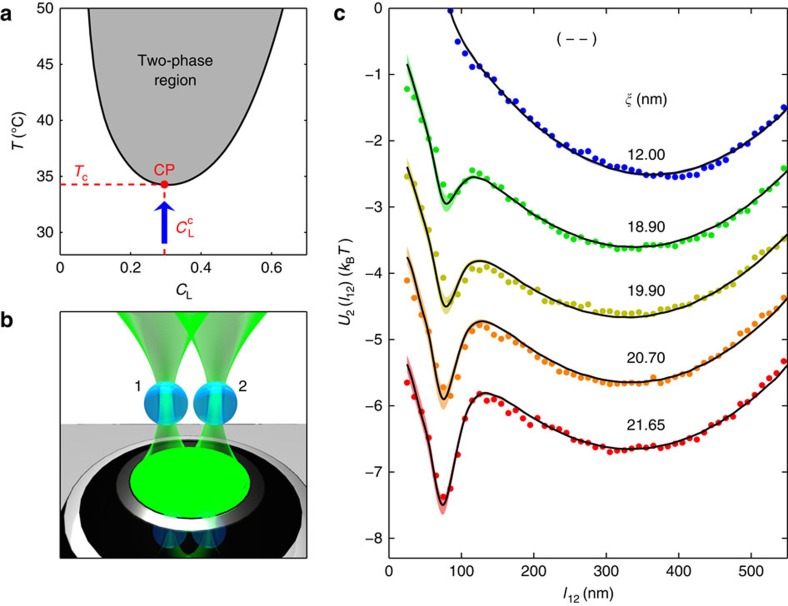
Two-body effective potentials in a critical mixture. (**a**) Phase diagram of the water–2,6-lutidine mixture featuring a lower critical point (CP) at the bottom of the coexistence line (thick solid line^39^). Measurements are performed at the critical lutidine mass fraction 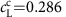, while the temperature *T* is gradually increased towards its critical value *T*_c_≃307 K, as indicated by the arrow. (**b**) Cartoon of the experimental set-up for the measurement of effective pair interactions: two spherical silica colloids (blue spheres, diameter 2*R*=2.06±0.05 μm) are held in the bulk of the binary mixture (not shown) by two optical tweezers (green conoids) obtained by focusing a laser beam via the objective indicated directly below (black and silver against a grey background). While the size of the particles is to scale with their relative distance, the objective and its distance from the particle are not. (**c**) Effective pair potential *U*_2_(*l*_12_) between two hydrophilic colloids labelled 1 and 2 (with boundary conditions (−−), that is, attractive critical Casimir forces) as a function of the in-plane surface-to-surface distance *l*_12_: the symbols represent the experimental data and the solid lines the theoretical fits with the associated uncertainty (shading). From top to bottom, *T* increases towards *T*_c_, which is accompanied by an increase of the fitted correlation length *ξ* and by the formation of an increasingly deep dip due to an attractive critical Casimir force, in agreement with the theoretical predictions. For clarity, symbols and curves corresponding to different temperatures have been separated vertically by a shift of 1 × *k*_B_*T*.

As solvent, we employ a mixture of water and 2,6-lutidine at the critical lutidine mass fraction 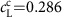 (see the phase diagram in Fig. 1a describing a lower critical point at the temperature *T*_c_≃307 K (refs 8, 9, 39)). A few degrees below *T*_c_, the mixture is homogeneous and critical Casimir forces are negligible. However, as *T* approaches *T*_c_ (arrow in Fig. 1a), critical concentration fluctuations emerge, which generate critical Casimir forces^7^. These forces depend strongly on the adsorption preferences of the surface of the particles, that is, on whether they preferentially adsorb water or lutidine, realizing (−) and (+) boundary conditions, respectively^8^,^9^. In particular, critical Casimir forces are attractive between two particles carrying the same boundary conditions, that is, (++) and (−−), while repulsive for (+−) and (−+). In the experiment, we employ both hydrophilic (−) pristine silica spheres (diameter 2*R*=2.06±0.05 μm) and hydrophobic (+) silica spheres obtained by treating their surfaces with octyltriethoxysilane.

We start by considering the configuration schematically shown in Fig. 1b, with two particles, labelled by 1 and 2. These particles are subject to a one-body potential *U*_ot_ due to optical traps, and a two-body contribution due to both a screened electrostatic pair repulsion *U*_es_ and a critical Casimir pair potential *U*_C_, while no evidence of van der Waals interactions was found; the total potential *U*_2_ of this configuration is therefore


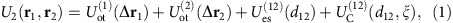


where **r**_*i*_ is the position of the centre of particle *i*, Δ**r**_*i*_=**r**_*i*_−**R**_*i*_, and **R**_*i*_ is the position of the centre of the *i*th trap, while *d*_*ij*_=|**r**_*i*_−**r**_*j*_|−2*R* is the distance between the surfaces of particles *i* and *j*. The optical traps are harmonic^40^, that is,


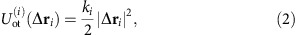


where the stiffness *k*_*i*_ (≃0.4 pN μm^−1^ for the data in Figs 1 and 4, and ≃0.8 pN μm^−1^ for those in Fig. 5) is nearly the same for all traps, as discussed below. The Derjaguin-Landau-Verwey-Overbeek (DLVO) electrostatic potential^1^ can be parameterised as


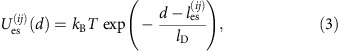


where *k*_B_ is the Boltzmann constant, *l*_D_ (≃10 nm) is the Debye length for the solvent and 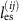 (≃90 nm) is a measure of the strength of the interaction between the two colloidal particles *i* and *j*, which depends, *inter alia*, on their surface charges. The mild dependence of both *U*_ot_ and *U*_es_ on the temperature *T* can be neglected, as here *T* is varied by at most 1%. Finally, the critical Casimir pair potential 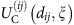 depends on the correlation length *ξ* of the critical mixture and, within the Derjaguin approximation 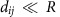 (with *d*_*ij*_≲0.3*R* in the present experiment), is given by^8^,^9^


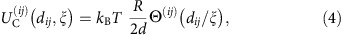


where Θ^(*ij*)^(*x*) is the universal scaling function characterized by the boundary conditions involved, but otherwise independent of the material properties of the mixture and of the particles; it can be inferred from available numerical data^8^,^9^,^41^,^42^. The correlation length *ξ* varies as *ξ*(*T*)=*ξ*_0_(1−*T*/*T*_c_)^−*ν*^, where the mixture-specific quantity *ξ*_0_=0.20±0.02 nm (s.e.m.) has been determined by light-scattering experiments^43^, while *ν*=0.63 is a universal critical exponent of the three-dimensional Ising universality class, holding for classical binary mixtures^9^. In the presence of three particles, their total potential *U*_3_ can be decomposed into the sum of individual one-body, pairwise two-body and remaining three-body contributions:


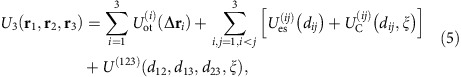


where 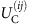 depends on the boundary conditions at the surfaces of the particles *i* and *j*, while *U*^(123)^ is the nonadditive three-body potential, which includes the contribution 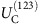 of the three-body critical Casimir potential, the existence and magnitude of which we want to assess. Note that 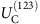 can be distinguished from other possible contributions (such as those due to electrostatics^2^,^3^) because of its sensitive dependence on temperature, which effectively controls also its spatial range.

### Measurement of two-body and three-body potentials

Until now direct measurements of critical Casimir forces have been performed only on single particles above planar surfaces using TIRM^8^,^10^; TIRM, however, necessarily involves a planar surface and therefore does not allow the measurement of forces arising between identical spherical particles, as they occur in colloidal suspensions. We have therefore developed an experimental set-up capable of manipulating and observing multiple particles in the bulk of a critical mixture. Our set-up is based on a combination of HOTs^34^,^40^ and DVM^35^,^36^,^40^; its schematic is shown in Supplementary Fig. 1. The HOTs are realised by shaping a laser beam using a spatial light modulator; in this way, we generate multiple reconfigurable optical traps within the sample, which allow us to gently hold the colloids with nanometric accuracy in the bulk of the critical mixture, that is, 50 μm above the lower surface of the sample cell; this distance is large enough to ensure that the critical Casimir forces between this surface and the particles are negligible. The laser power at each trapping site is kept low enough (≃2 mW) to avoid significant heating (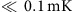 (ref. 40)). The DVM uses a monochromatic camera acquiring videos at 200 fps; these frames are analysed using standard DVM algorithms to determine the projected positions of the particles with nanometric accuracy^35^,^36^ (see Methods for details). The crucial temperature control of the binary mixture is achieved in two stages as described in the Methods section and is able to maintain a set temperature with an accuracy of ±2 mK.

**Figure 2:**
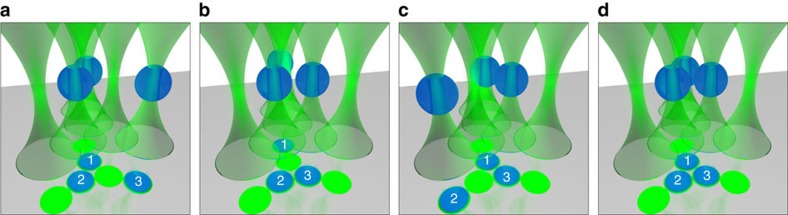
Experimental configurations. Cartoon of the geometrical arrangement of the six optical tweezers (green conoids) and the colloids (blue spheres) during the experiment. The resulting harmonic optical traps are arranged as indicated beneath by the schematic orthogonal projection on the coverslip, with colloids and traps represented by numbered blue and dark green circles, respectively. The sizes of the particles are to scale with their distance, but not with the distance from the coverslip. At each temperature *T*, the interactions between the pairs 1–2, 1–3 and 2–3 of colloids are measured in the configurations **a**,**b** and **c**, respectively. The effective potential between colloids 1 and 2 in the presence of colloid 3 is then measured in the configuration **d**. While measuring the pair interactions in **a**–**c**, the remaining colloid is optically moved into the nearest external trap. All six optical traps are always switched on during the measurements in order not to alter the hologram on the spatial light modulator and the corresponding optical potentials.

We perform all measurements using the same configuration of six traps, all obtained by means of the same hologram on the spatial light modulator, which produces spatially displaced but otherwise almost identical optical potentials. Three of these traps, referred to as internal ones, have their centres located at the vertices of an approximately equilateral triangle with edges of ≃2.3 μm; the centres of the remaining three traps, referred to as external ones, are instead located at a distance ≃2.3 μm from each vertex, along the bisector of the corresponding external angle (Fig. 2). The six traps are sufficiently far apart to guarantee the independence of the optical potentials^40^. The internal traps are used to hold either two (Fig. 2a–c) or three particles (Fig. 2d) for measuring the pair interaction or three-body potentials, respectively. The particles that are not needed in a certain measurement are temporarily moved from the internal to the closest of the external traps, where they have negligible interactions with the other particles. There, their surface-to-surface distance is >100 times larger than both the largest value of the range *ξ* of the critical Casimir forces and the typical range *l*_D_ of the electrostatic repulsion involved in the experiment. In this way, the three colloids—labelled 1, 2 and 3—are selected at the beginning of the experiment and employed for the whole set of measurements; this eliminates possible systematic errors due to differences in the properties of the colloids of the batch we use. We start at a temperature *T* a few degrees below *T*_c_, for which no critical fluctuations are present and the mixture is homogenous. First, we characterise the optical potential in equation (2) by measuring *k*_*i*_ of each internal trap employing the configurations in Supplementary Fig. 2a: the particle under measurement (for example, particle 1 in configuration a1) is held in an internal trap, while the other two particles (for example, particles 2 and 3 in configuration a1) are moved into the nearest external traps. Then, by holding each pair (that is, 1–2, 1–3 and 2–3) of particles in the respective internal traps while keeping the third particle (that is, 3, 2 and 1, respectively) in the corresponding external trap, we measure and characterise the electrostatic interaction in equation (3) between each possible pair of particles, using the configurations shown in Fig. 2a–c, respectively. Finally, we measure the three-body interactions by having all three colloids in the internal traps as shown in Fig. 2d. At temperatures much lower than *T*_c_, we obtain additivity of the interactions (in particular, of the electrostatic one), as expected. We then increase *T* in small steps towards *T*_c_ and repeat the measurement of the pair and three-body interactions at each step. The details of the measurement cycle are shown in Supplementary Fig. 2b.

**Figure 3:**
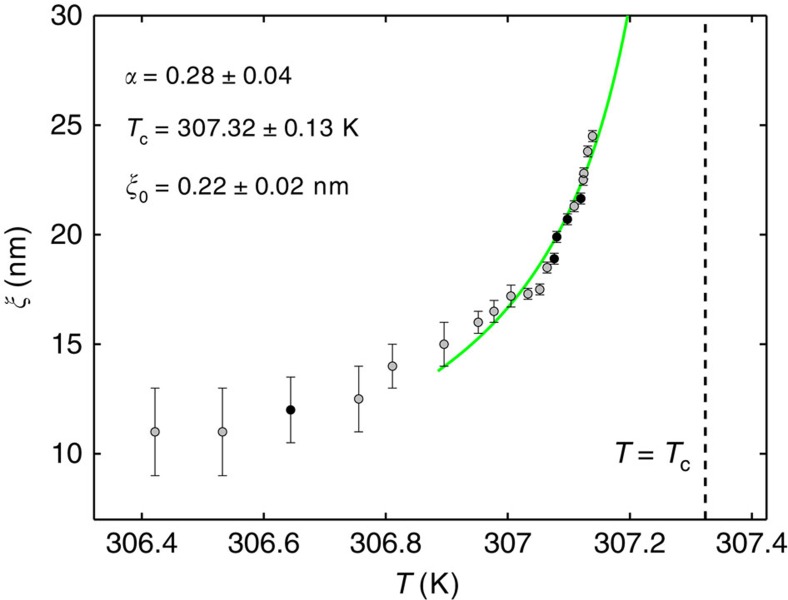
Temperature dependence of the correlation length. The symbols indicate the values of the bulk correlation length *ξ* obtained from the best fit to the experimental data for the effective pair potential *U*_2_(*l*_12_); the error bars represent 2 s.d.'s. The black symbols correspond to the data displayed in Fig. 1c. To compare with the theoretical prediction given by equation (4), we take into account that the actual temperature *T* of the mixture is expected to be a linear combination of the temperature *T*_o_ set at the microscope objective, which is varied during the experiment, and the fixed temperature *T*_b_=306.15 K of the thermal bath, that is., *T*=(1−*α*)*T*_b_+*αT*_o_ with 0<*α*<1. The green solid line corresponds to the best fit of the expected algebraic law *ξ*=*ξ*_0_(1−*T*/*T*_*c*_)^−*ν*^ with *ν*=0.63 for the data with *ξ*≳15 nm, which yields *α*=0.28±0.04, *T*_c_=307.32±0.13 K and *ξ*_0_=0.22±0.02 nm. This estimate of *ξ*_0_ is compatible with the results of light-scattering experiments (see, for example, Table III in ref. 9). The estimate of *T*_c_ (indicated by the vertical dashed line) agrees with the known value for water and 2,6-lutidine. The overall agreement between the experimental data and the expected behaviour is quite good.

For each value of *T*, we acquire the histogram of the probability distribution *P*_2_(*l*_12_) of the in-plane surface-to-surface distance *l*_12_ between the particles 1 and 2 (see Methods for the definition of *l*_12_), from which we infer the effective potential *U*_2_(*l*_12_)≡−*k*_B_*T* ln *P*_2_(*l*_12_) shown by the symbols in Fig. 1c for various values of the correlation length *ξ* determined as described below. The solid lines in Fig. 1c represent, instead, the theoretical predictions based on equation (1) obtained via a Monte Carlo integration of the Boltzmann factor exp(−*U*_2_(**r**_1_, **r**_2_)/(*k*_B_*T*)) (see Methods for additional details), where the only fitting parameter is *ξ* because the optical and the electrostatic potentials have already been characterized at low *T* (see above). Note that the indicated values of *ξ* have been determined by a best fit to a part of the experimental data (highlighted by darker colours in the histograms in Supplementary Fig. 3) and are the same for the three pairs of particles as *ξ* only depends on the temperature of the mixture; the relationship between the sample temperature *T* and the correlation length is shown in Fig. 3. We obtain a very good agreement between the measured and the theoretical effective potentials for all three pairs of particles (Supplementary Fig. 3a–c). This demonstrates that equation (4) properly describes the two-body critical Casimir interaction 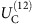, which is responsible for the formation of the dip in *U*_2_(*l*_12_) at *l*_12_≃80 nm and which appears as *ξ* increases. In addition, this agreement provides the direct experimental evidence of the occurrence of critical Casimir forces between two spherical colloidal particles, which is a geometrical configuration that had not been previously explored. Note that *U*_2_(*l*_12_) at separations *l*_12_≳250 nm is essentially determined by *U*_ot_, while for *l*_12_≲50 nm it is strongly influenced by the short-distance behaviour of *U*_es_, which might not be accurately captured by equation (3). Accordingly, the range of *l*_12_ relevant for assessing the comparison between theory and experiment, and the emergence of (possibly many-body) critical Casimir forces, extends from the bottom of the dip to its right.

**Figure 4:**
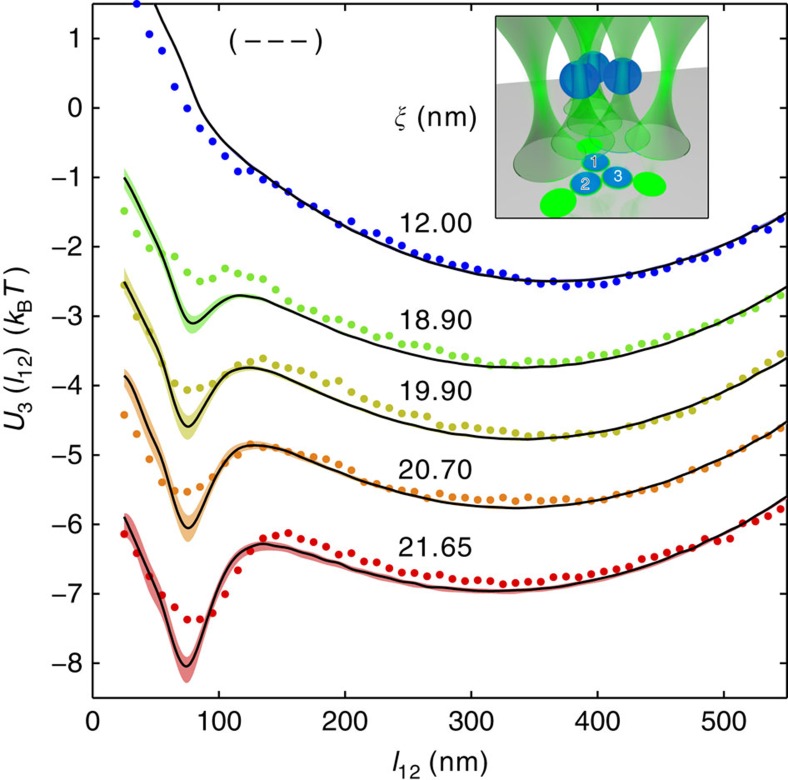
Many-body critical Casimir forces with symmetric boundary conditions. The symbols represent the measured effective potential *U*_3_(*l*_12_) between the particles 1 and 2 (labelled in black in the inset) in the presence of particle 3 (labelled in white in the inset) as a function of the in-plane surface-to-surface distance *l*_12_ on increasing (from top to bottom) the correlation length *ξ*. All particles are hydrophilic (−−−), resulting in attractive critical Casimir forces. The solid lines represent the corresponding theoretical predictions obtained by assuming additivity of the measured pair potentials between particles 1–2, 1–3 and 2–3 with the associated uncertainty indicated by the shading. The observed discrepancy increases as *ξ* increases, providing quantitative evidence of the nonadditive nature of the critical Casimir interactions. The colour code of the data points is the same as in Fig. 1, and symbols and lines are vertically separated by 1 × *k*_B_*T* for reasons of clarity. Inset: cartoon of the trap and colloid configuration during the measurement (see Fig. 2 for details).

At this point, we can predict the effective potential *U*_3_(*l*_12_) associated with the distribution *P*_3_(*l*_12_) of the in-plane distance between particles 1 and 2 in the presence of particle 3 (Fig. 2d) using the measured two-body interactions and assuming additivity, that is, *U*^(123)^≡0 in equation (5). Again, these theoretical predictions are computed numerically via a Monte Carlo integration of the Boltzmann factor exp(−*U*_3_(**r**_1_, **r**_2_, **r**_3_)/(*k*_B_*T*)) (see Methods for additional details). The resulting effective potential *U*_3_(*l*_12_) between particles 1 and 2 is indicated by the solid lines in Fig. 4, while the symbols are the corresponding experimental data: the clear discrepancy between the two provides evidence for the presence of nonadditive (many-body) effects. The data with the smallest *ξ* depart appreciably from additivity at short distances (*l*_12_≲70 nm) due to a short-ranged electrostatic three-body effect that reduces repulsion^3^ and therefore amplifies the effects of the two-body critical Casimir attraction. On the contrary, as *ξ* increases, the effective potential is less attractive than expected, indicating that such a reduction is due to the many-body critical Casimir interaction 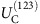, especially for *l*_12_ between 70 and 250 nm, where the two- and three-body electrostatics effects are negligible.

**Figure 5:**
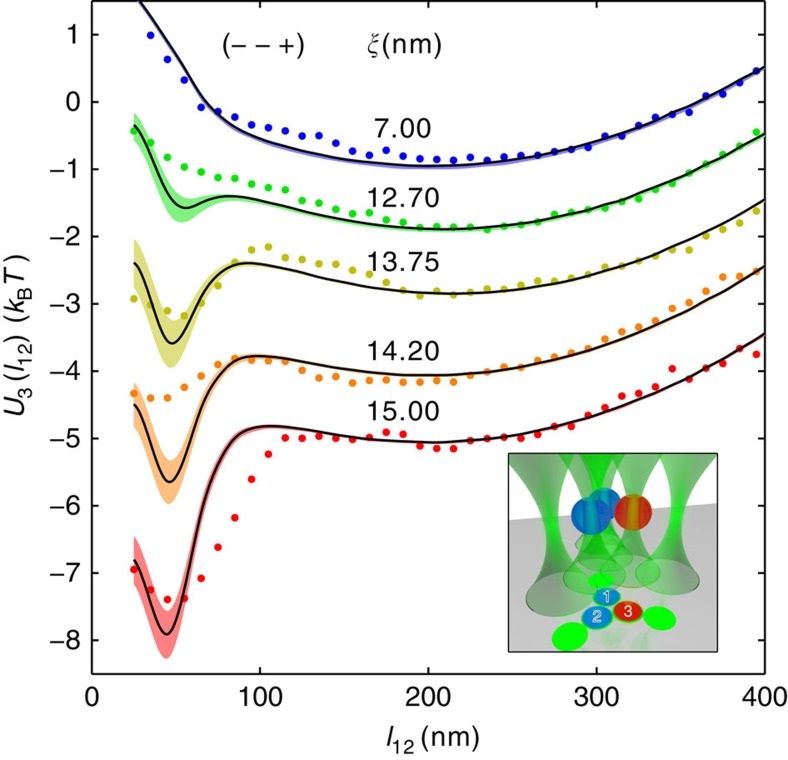
Many-body critical Casimir forces with asymmetric boundary conditions. The symbols represent the measured effective potentials *U*_3_(*l*_12_) between particles 1 and 2 (blue, labelled in black in the inset) in the presence of particle 3 (red, labelled in white in the inset) as a function of the in-plane surface-to-surface distance *l*_12_ on increasing (from top to bottom) the correlation length *ξ*. Particles 1 and 2 (blue spheres in the inset) are hydrophilic (−), while particle 3 (red sphere in the inset) is hydrophobic (+), so that the two-body critical Casimir forces cause attraction between 1 and 2 and repulsion between 2 and 3, and between 3 and 1. The solid lines represent the corresponding theoretical prediction obtained by assuming additivity of the measured pair potentials between particles 1–2, 1–3 and 2–3, with the associated uncertainty indicated by the shading. The observed discrepancy between the lines and the symbols increases as *ξ* increases, providing quantitative evidence of the nonadditive nature of critical Casimir interactions also in the presence of opposing boundary conditions. Symbols and lines corresponding to different temperatures are vertically separated by 1 × *k*_B_*T* for reasons of clarity. Inset: cartoon of the trap and colloid configuration during the measurement (see Fig. 2 for details).

As pointed out above, one of the most distinguished features of the two-body critical Casimir forces is that their attractive or repulsive character depends on the surface properties of the particles. While the experiment described above involves three hydrophilic particles (−−−), we repeated the experiment with one hydrophobic (3) and two hydrophilic (1, 2) particles (−−+); the corresponding results are shown in Fig. 5 and Supplementary Fig. 4. In this experimental setting, the distance between the internal traps is slightly decreased by ≈0.1 μm; consequently, the electrostatic repulsion between the trapped particles increases and thus a trap twice as stiff is required to keep the colloids sufficiently close to experience critical Casimir forces at correlation lengths similar to those explored in the (−−−) configuration. Also in this case, we observe good agreement between theory and experiment for the pair-interaction effective potentials *U*_2_(*l*_*ij*_) that can be inferred from the histograms of the corresponding distribution *P*_2_(*l*_*ij*_) (Supplementary Fig. 4a–c), while sizeable discrepancies emerge in the three-body potential *U*_3_(*l*_12_) (Fig. 5; Supplementary Fig. 4d). In contrast to the previous case, the experimental data demonstrate that, depending on the distance and the correlation length, the many-body effects may also deepen the critical Casimir potential between particles 1 and 2.

The experiments described above typically correspond to values of the scaling variables *R*/*ξ*≃100 and *l*_*ij*_/*R*≃0.1 that are outside the ranges of the available theoretical predictions for the many-body effects^32^: their highly nontrivial dependence on temperature and geometrical features renders questionable any attempt to extrapolate and therefore to compare these predictions with the present experimental data. Interestingly enough, assuming for 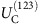 the simple functional form of the Axilrod-Teller three-atom potential that describes three-body corrections to the van der Waals interaction (see, for example, ref. 32), with a suitable choice of the overall amplitude, reduces the discrepancy between the experimental and the corresponding theoretical predictions.

## Discussion

Our results provide direct experimental evidence of the emergence of critical Casimir forces between two colloids and demonstrate the presence of pronounced three-body effects. These many-body critical Casimir forces strongly depend on the proximity to criticality of the fluid solvent, and can therefore be tuned, for example, by changing the temperature of the system or by altering the surface properties of the involved colloids. While we have focused on configurations with three particles, the experimental set-up and protocol discussed here are versatile enough to allow the investigation of the many-body potentials associated with a larger number of particles, possibly of non-spherical shape and dissolved in a variety of different solvents. In particular, in salty near-critical mixtures, one can expect a subtle interplay between the many-body effects of electrostatic and critical Casimir forces similar to those observed in the case of the two-body interaction between charged colloids^44^,^45^. Anisotropic solvents such as nematic liquid crystals, instead, may lead to effective many-body interactions with a complex dependence on the direction of the nematic director^46^ and on the shape of the involved colloids^47^, which could be investigated by extending the present approach. Criticality amounts to the occurrence of order parameter fluctuations on the spatial scale of the correlation length, which in principle can diverge and, thus, lead to the emergence of complex and nonadditive interactions at very large scales. They may find natural applications in various disciplines, such as in the realization of colloidal molecules or reversible self-assembly, as well as in the organization of cellular membranes^48^ and possibly even in the patterns of brain activation^49^. Furthermore, in view of their similarity, these effects can also shine light on certain aspects of the many-body effects in QED Casimir forces, while the experimental approach described here can be naturally extended to the recent attempt to measure these forces with optical tweezers^50^.

## Methods

### Experimental set-up

The experimental set-up combines HOTs and DVM (Supplementary Fig. 1), and is build around a home-made microscope. A laser beam (wavelength *λ*=532 nm, power 500 mW) is expanded by a telescope and projected onto a phase-only spatial light modulator (Holoeye, PLUTO-VIS). The hologram on the spatial light modulator imposes a phase modulation onto the beam, allowing the generation of multiple trapping spots. The resulting beam is then projected onto the entrance pupil of a high-numerical-aperture oil-immersion objective (magnification × 100, numerical aperture 1.30) by a series of lenses and mirrors arranged in a 4f configuration^40^. The objective focuses the beam in the sample plane and creates a series of reconfigurable traps. As the mixture approaches criticality, the induced fluctuations of the refractive index may in principle interfere with HOT and alter the features of the resulting optical potentials. However, the correlation length *ξ*≲22 nm explored in our experiments (Fig. 3) remains always sufficiently small in comparison with *λ*, and thus does not significantly influence the optical beam propagation and, therefore, the generated optical potentials. In particular, the values of the trap stiffnesses *k*_*i*_, which we infer from the best fits to the experimental data, show no systematic dependence on *T*. Since the configuration on the phase mask imposed on the spatial light modulator is always the same during each data acquisition (Supplementary Fig. 2), the resulting optical trapping potentials are also always the same. In addition, during the calibration of the set-up, we have tested the stability of all internal and external traps with particles in the configurations used later while acquiring the data; this allowed us to confirm the harmonicity of the traps and to exclude the presence of ghost traps in the region of space explored by the particles. The DVM is realised using a standard configuration with white light illumination and a monochromatic CMOS camera (200 f.p.s.). Since, near *T*_c_, critical fluctuations depend very sensitively on small temperature changes, the microscope is enclosed within a thermally stabilised box to avoid any air flow, which may cause instability of the sample temperature. The sample holder is thermally isolated from the underneath translation stage with a teflon film. The necessary fine control of the temperature of the sample is achieved in two stages: first, the temperature of the microscope box is controlled to within ±50 mK; second, a closed-loop controller (realized with a Pt100 temperature sensor and a Peltier heating/cooling element) keeps the temperature of the sample cell to within ±2 mK. We remark that within each set of experiments the temperature *T* is gradually increased towards *T*_c_.

### Sample preparation

The binary liquid mixture used in all experiments is composed of water and 2,6-lutidine at the critical lutidine mass fraction 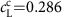. The mixture undergoes a second-order phase transition at a lower critical point with temperature *T*_c_≃307 K (refs 8, 9, 39). The corresponding phase diagram is shown in Fig. 1a (ref. 39): it consists of two regions, corresponding to the mixed (white) and the demixed (grey) phases with a lower critical point. In all experiments, we use silica colloids with diameter 2*R*=2.06±0.05 μm (Microparticles GmbH). In the measurements involving hydrophobic particles, these silica colloids are treated chemically to make them hydrophobic, that is, we silanized their surfaces with octyltriethoxysilane. The sample cell containing the critical mixture and a small amount of colloids is a 200-μm-thick silica cuvette, which is sealed with teflon plugs to avoid evaporation of the mixture and to allow its usage for the whole duration of the experiment (≈1 day).

### Data analysis

The raw data obtained from the various measurements are videos showing the orthogonal projection on a plane of the Brownian motion of the particles in three spatial dimensions and consisting of ≈60,000 frames acquired at 200 f.p.s. From these videos, we extract the in-plane position (*x*_*i*_ and *y*_*i*_) of the centre of each colloid *i* as a function of time using standard DVM algorithms^35^,^40^. During the measurements of two- and three-body potentials, the surface-to-surface distance between the involved colloids is typically smaller than their common radius *R*; accordingly, to obtain the actual value *r*_phys_ of the distance between their centres, it is necessary to correct^36^ the measured optical distance *r*_meas_ by adding a distance-dependent correction *δr*(*r*_meas_). We have determined the function *δr*(*r*_meas_), which approximately ranges between −60 and −10 nm in our experiments, on the basis of the optical images of single colloids, as explained in ref. 36. On the basis of these trajectories, we calculate the in-plane surface-to-surface distance 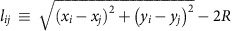 between particles *i* and *j*, which allows us to construct the histograms *P*_*n*_(*l*_*ij*_), corresponding to the acquired frames with *n*=2 or 3 particles being close, and from them to determine the effective potentials *U*_*n*_(*l*_*ij*_)=−*k*_B_*T* ln *P*_*n*_(*l*_*ij*_). Since *l*_*ij*_ is obtained from a differential measurement, all common-mode noise (for example, due to slow mechanical drifts over the duration of the experiment) is automatically removed.

### Monte Carlo integration

For each set of parameters, we compute the theoretical in-plane surface-to-surface separation histograms *P*_*n*_(*l*_*ij*_) and the associated effective potentials *U*_*n*_(*l*_*ij*_) via a suitable Monte Carlo integration of the Boltzmann factors exp(−*U*_2_(**r**_1_, **r**_2_)/(*k*_B_*T*)) and exp(−*U*_3_(**r**_1_, **r**_2_, **r**_3_)/(*k*_B_*T*)) with the theoretical potentials given by equations (1), (4) and (5) with *U*^(123)^=0 for the configurations with two and three close particles, respectively, based on the measured parameters of the optical traps and of the electrostatic interaction (equation (3)). For instance, in the presence of three close particles, one has 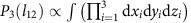 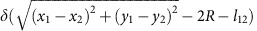

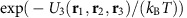
, where **r**_*i*_≡(*x*_*i*_, *y*_*i*_, *z*_*i*_) is the position of particle *i* in a Cartesian coordinate system. To account for a (small) anisotropy of the generated optical traps, the data analysis and the fit are carried out by assuming 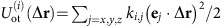, where **e**_*x*,*y*,*z*_ are the unit vectors along the principle orthogonal axes of the ellipsoidal trap, instead of equation (2). For all traps, **e**_*x*,*y*_ turn out to lie almost within the *x*−*y* plane, with *k*_*i,x*_≃*k*_*i,y*_ and *k*_*i,z*_≃0.3*k*_*i,x*_. In particular, we verified that the actual values of *k*_*i*,*z*_ do not significantly affect the comparison between the theoretical predictions and the experimental data. The Monte Carlo integration discussed here is necessary because of the large number of variables (6 and 9 for *P*_2_ and *P*_3_, respectively) over which the Boltzmann factor has to be integrated. The presence of the harmonic optical traps is exploited to introduce an importance sampling based on the corresponding Gaussian distributions, assumed to have variances ≃*k*_B_*T*/*k*_*i,x*_, *k*_B_*T*/*k*_*i*,*y*_ and *k*_B_*T*/*k*_*i*,*z*_ along the three spatial axes. We have checked that the eventual theoretical predictions for both the two- and three-body potentials are not affected by this assumption or by the number of sampled points in space, which was set to a sufficiently large value, of the order of 10^7^−10^8^.

## Additional information

**How to cite this article:** Paladugu, S. *et al*. Nonadditivity of critical Casimir forces. *Nat. Commun.* 7:11403 doi: 10.1038/ncomms11403 (2016).

## Supplementary Material

Supplementary Information Supplementary Figures 1-4
