# Supplementary material for: Nonadditivity of critical Casimir forces
Source: Nat Commun. 2016 Apr 21;7:11403. doi: 10.1038/ncomms11403 (PMC4844688; doi:10.1038/ncomms11403)
Supplement: Supplementary Information — Supplementary Figures 1-4 [file ncomms11403-s1.pdf]

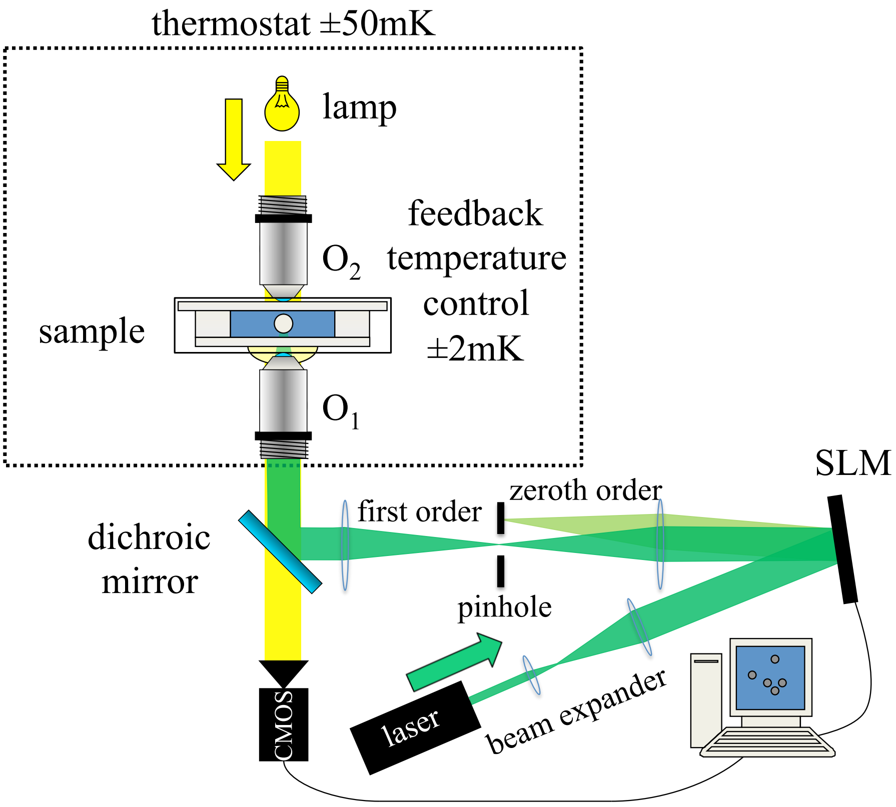

Supplementary Figure 1 | Experimental setup. The experimental setup consists of a holographic optical tweezers (HOTs) and a digital video microscope (DVM). The former generates multiple reconfigurable optical traps by imposing a phase-only hologram on an incoming beam using a spatial light modulator (SLM) and by focusing the resulting beam with a high-numerical-aperture objective (O_1). The latter tracks the position of the colloids within the horizontal xy-plane with nanometric accuracy by illuminating the sample with white light through a condenser (O_2). A dichroic mirror (DM) is employed in order to combine the optical paths of the laser and of the white light. The temperature of the sample is controlled as explained in the Methods and as indicated in the schematic of the setup.

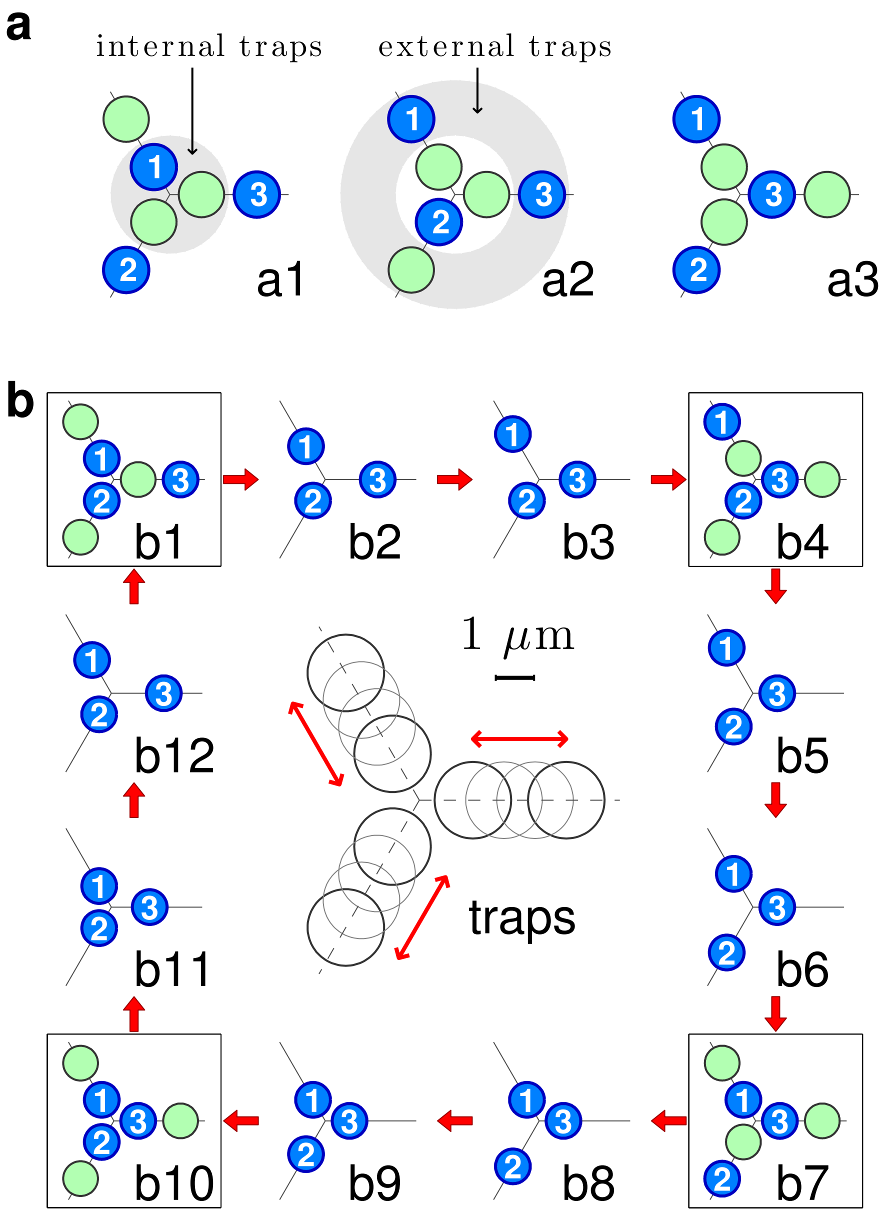

Supplementary Figure 2 | Particle configurations and protocol to switch between them. **a**, Particle configurations for the characterisation of the one-body optical potential associated with each optical trap. Empty and occupied optical traps are represented by green and blue circles, respectively. **b**, Protocol to switch between the configurations (framed) in which the measurements are performed: at each temperature, first the two-body interactions between the pairs 1-2, 1-3 and 2-3 of colloids are measured in the configurations b1 (Fig. 2a), b4 (Fig. 2b) and b7 (Fig. 2c), respectively; then, the effective potentials between the three colloids is measured in the configuration b10 (Fig. 2d). For rearranging the colloids in these configurations, the intermediate optical traps indicated by thin grey circles in the central sketch (with the associated length scale) are switched on and off in order to move the particles from the internal to the external traps and vice versa, realising the intermediate configurations indicated in the panel.

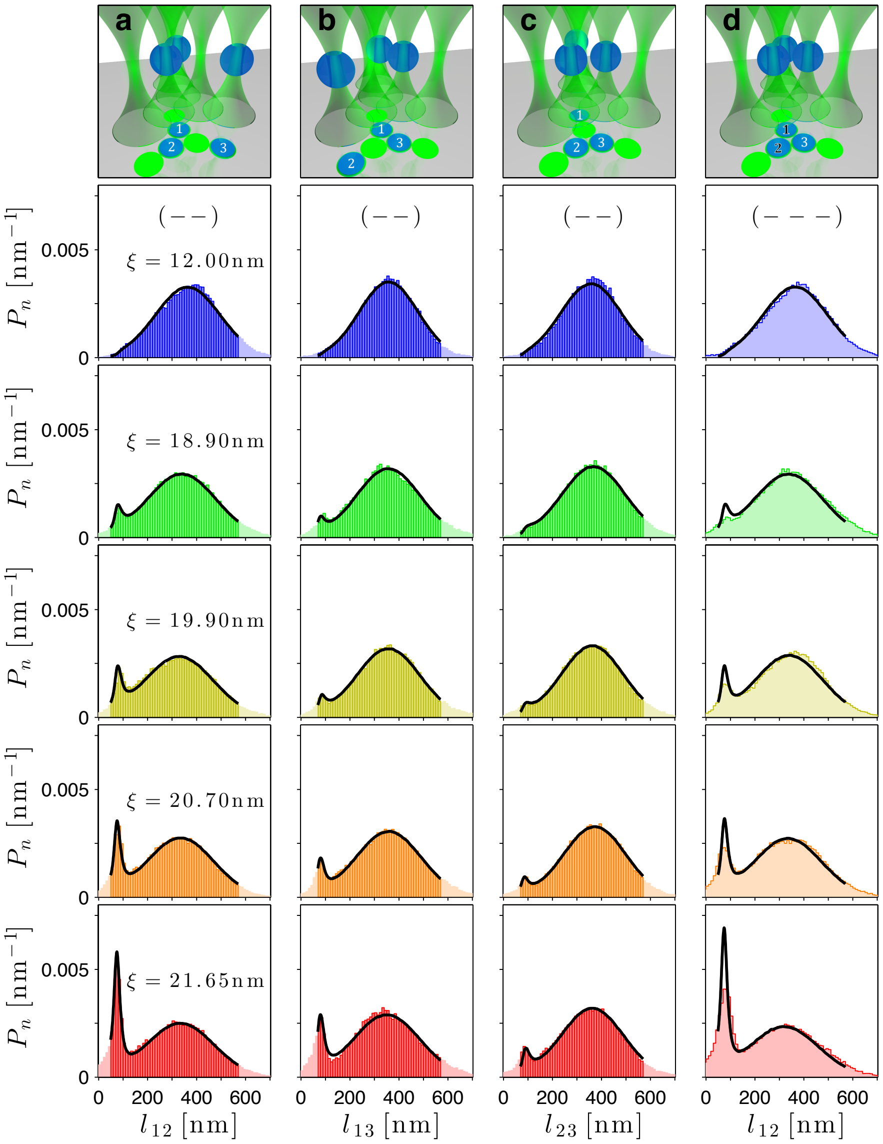

Supplementary Figure 3 | Interparticle separation histograms for symmetric boundary conditions. **a-c**, Histograms $P_2(l_{ij})$ of the in-plane surface-to-surface distance l_{ij} between particles 1-2, 1-3 and 2-3, shown in columns **a**, **b** and **c**, respectively, for increasing values of the correlation length ξ, from top to bottom; the values given for ξ hold for the whole corresponding line of panels. The solid lines represent fits to the theory corresponding to Eq. (1), which show very good agreement. For each row of plots, ξ was determined by the best global fit to the experimental data for $P_2(l_{ij})$ highlighted by darker colours in the two-particle configurations **a**, **b** and **c**. The remaining parameters were already determined at low temperatures, at which the critical Casimir potential is negligible. **d**, Histograms $P_3(l_{ij})$ of the in-plane surface-to-surface distance l_{12} between particle 1 and 2 (labelled in black) in the presence of particle 3 (labelled in white). The solid lines indicate the theoretical prediction assuming additive critical Casimir forces and show a clear discrepancy (mainly at the left peak) with the experimental histograms, which increases upon increasing ξ. The histograms in columns **a** and **d** correspond to the effective potentials reported in Figs. 1c and 4, respectively. First row: Cartoon of the trap and colloid configurations during the measurement (see Fig. 2 for details).

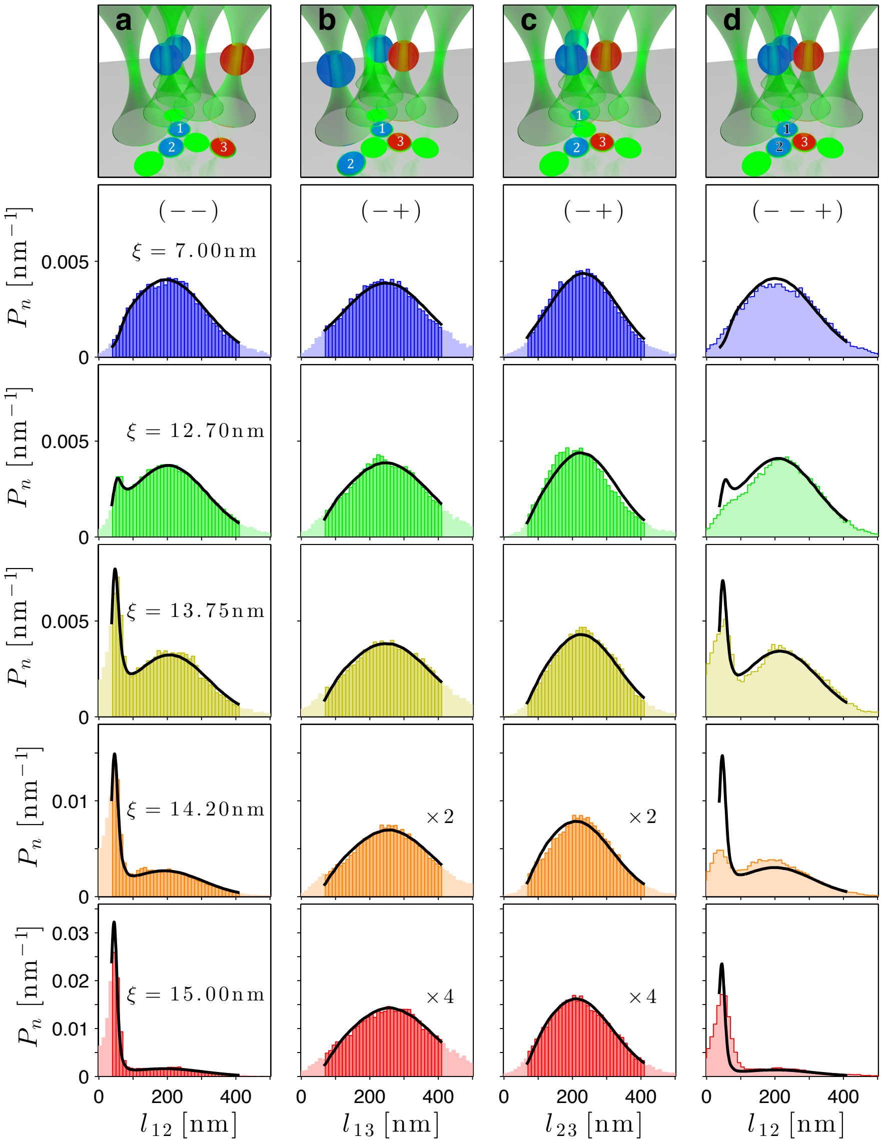

Supplementary Figure 4 | Interparticle separation histograms for asymmetric boundary conditions. **a-c**, Histograms $P_2(l_{ij})$ of the in-plane surface-to-surface distance l_{ij} between particles 1-2 (−−), 1-3 (−+) and 2-3 (−+), shown in columns **a**, **b** and **c**, respectively, for increasing values of the correlation length ξ, from top to bottom; the values given for ξ hold for the whole corresponding line of panels, while the data in the two bottom panels of columns both **b** and **c** have been magnified by the indicated factors in order to be on the same scale as the other panels. The solid lines represent fits to the theory corresponding to Eq. (1), which are very good. As in Supplementary Fig. 3, ξ was determined for each row of plots by the best global fit to the experimental data for $P_2(l_{ij})$ highlighted by darker colours in the two-particle configurations **a**, **b** and **c**. The remaining parameters were already determined at low temperatures, at which the critical Casimir potential is negligible. Note that the histograms in columns **b** and **c** do not feature the emergence of a pronounced peak (corresponding to an attractive dip in the effective potential) because the critical Casimir pair potential with (−+) boundary conditions is repulsive. **d**, Histograms $P_3(l_{12})$ of the in-plane surface-to-surface distance l_{12} between particles 1 and 2 (−−, blue spheres) in the presence of particle 3 (+, red sphere). The solid lines show the theoretical predictions assuming additive critical Casimir forces; there are clear discrepancies with the experimental histograms, which become more pronounced upon increasing ξ. The histograms in **d** correspond to the effective potentials reported in Fig. 5. First row: Cartoon of the trap and colloid configurations during the measurement (see Fig. 2 for details), with hydrophilic and hydrophobic particles represented as blue and red spheres, respectively.
